# Common brain representations of action and perception investigated with cross-modal classification of newly learned melodies

**DOI:** 10.1038/s41598-025-00208-x

**Published:** 2025-05-12

**Authors:** Yu-Hsin Fiona Chang, Fredrik Ullén, Örjan de Manzano

**Affiliations:** 1 Department of Cognitive Neuropsychology, Max Planck Institute for Empirical Aesthetics, Grüneburgweg 14, 60322 Frankfurt, Germany; 2 Department of Neuroscience, Karolinska Institutet, Stockholm, Sweden

**Keywords:** Action-perception coupling, fMRI, Music perception, MVPA, Sequence learning, Perception, Human behaviour, Cortex

## Abstract

An important feature of human cognition is the ability to predict sensory outcomes of motor actions and infer actions from sensory information – a process enabled by action-perception coupling. Through repeated and consistent sensory feedback, bidirectional sensorimotor associations can become highly automatic with experience. In musicians, for instance, auditory cortex activity can increase spontaneously even when observing piano playing without auditory feedback. A key question is whether such associations rely on shared neural representations, or a “common code”, between actions and their sensory outcomes. To test this, we trained non-musicians to play two melodies with different pitch sequences on the piano. The following day, they underwent an fMRI experiment with an MR-compatible piano while (a) *playing the trained melodies without auditory feedback but imagining the sound*, and (b) *listening to the same melodies without playing but imagining the finger movements*. Within-condition multivariate pattern analyses revealed that patterns of activity in auditory-motor regions represent pitch sequences. Importantly, cross-modal classification showed that these patterns generalized across conditions in the right premotor cortex, indicating the emergence of a common code across perception and action.

## Introduction

Motor actions are often associated with sensory consequences that we learn to anticipate. For example, when learning to play the piano, we become familiar with the sounds that result from pressing down different piano keys. The process of establishing such associations between actions and their sensory outcomes is referred to as action-perception coupling^1^. A long-standing question in cognitive neuroscience is how our brain performs such sensorimotor integration. The ideomotor theory states that there are bidirectional associations between movements and their sensory consequences, and that an action can be initiated by representing the desired perceptual effect which in turn triggers the motor program required to reach that goal state. The common coding principle extends this idea by proposing a common representational “code” for both action and perception at higher levels of abstraction^2–4^. However, such a common code has yet to be demonstrated.

Musical performance has proved an excellent model for exploring action-perception coupling due to the required strong integration between auditory and motor systems. Through extensive training, musicians develop strong bidirectional associations between finger movements and auditory feedback from their musical instruments. During performance, movements are guided by the intended auditory outcomes, and conversely, listening to melodies may prime the associated movements^5–9^. Behavioral studies have shown that experimentally induced incongruences between actions and expected sounds interfere with musicians’ performance but not that of non-musicians, highlighting the strength of these learned sensorimotor associations^10^. Neuroimaging studies provide similar findings: Haslinger et al.^11^, for instance, found that when observing silent piano playing, pianists – compared to musically naïve participants – exhibited higher activity not only in the premotor cortex but also auditory cortex. This could be interpreted as action observation triggering representations of sounds in the “mind’s ear” of the musicians. In the reverse direction, Lahav and colleagues showed that after brief piano training, non-musicians had additional activity in the premotor cortex when listening to the learned melody but not when listening to novel music^12^. All these findings highlight the importance of training for establishing sensory-motor associations.

An additional important advantage of action-perception coupling is that it enables imagery, i.e. mental simulation. Musicians often engage in mental rehearsal, which involves imagining the movements and sensory experiences associated with playing the instrument, thereby presumably activating the neural circuits associated with organizing motor output. Similarly, visualizing actions and outcomes is considered vital preparation for optimal performance in many sports^13–15^. Interestingly, research has shown that even in the absence of actual physical experience, mental imagery can activate the corresponding neural substrate. This is supported by brain imaging studies that find overlapping areas of brain activity during motor execution and motor imagery^16,17^. Furthermore, studies on auditory processing and motor imagery reveal substantially shared neural substrates^18–20^.

Most of the studies to date have primarily demonstrated a spatial overlap of neural activity between observation, imagery, and motor execution^21^. A more specific question, however, is whether these activations also represent a content-specific overlap, i.e. shared neural representations, or a “common code”, as mentioned previously. Multivariate pattern analysis (MVPA) can address this by using machine learning to determine, for instance, whether a classifier can learn to differentiate between voxel-wise patterns of brain activity that correspond to different mental content^22,23^. The advantage of MVPA in comparison to traditional univariate analysis, is that the informational content of brain activity is not left to interpretation, but can be probed and confirmed with a machine learning classifier that is first trained, and then tested on previously unseen data. If the classification can be done above chance with for instance permutation testing, even in a few individuals, this is strong evidence that spatial patterns of activity in a given brain region can indeed encode the labels provided. Notably, MVPA is not limited to decoding information within the same modality, but can also provide evidence for the generalization of representations across modalities^24,25^. For instance, Etzel, et al.^26^ trained a classifier on brain activity patterns associated with the motor execution of hand or mouth actions, then tested whether it could classify activity induced by listening to the corresponding action sounds. They found that the classifier was able to decode the information in the premotor cortex across modalities. This provides a convincing case that the same representations were used both in performance and perception.

Thus far, the majority of evidence supporting the common coding principle has been limited to single actions^27,28^. However, many – if not most – of our daily tasks are composed of numerous individual actions carried out continuously in sequential order. It is therefore crucial to understand how the brain represents sequences of actions. Using MVPA, several studies have revealed the representations of sequential movements in motor and parietal regions^29–31^. In our previous work, we also applied MVPA to test if a classifier was able to differentiate between brain patterns associated with listening to different pitch sequences (i.e. melodies), which non-musicians had learned to play on the piano^32^. Consistent with the ideomotor theory, our findings indicated that perceiving learned sequences elicited sequence-specific representations not only in the auditory cortex but also in the premotor cortex, presumably through action-perception coupling. This did not happen in a control group that listened to melodies without piano training, which confirms the training-dependent nature of these sensorimotor associations.

In the present study, we aimed to extend this finding to also test the common coding principle. Specifically, we hypothesized that the same neural representations would be recruited during piano playing without auditory feedback (while imagining the sound), as during listening to the same trained musical material without overt movements (while imagining playing).

For this purpose, we designed an experiment in which participants without prior musical training learned to play two melodies on the piano. On the subsequent day, an fMRI experiment was performed in which the participants (a) *played the melodies without sound while imagining the auditory output* (playing condition), and (b) *listened to the same melodies without playing while imagining the corresponding finger movements* (listening condition). We first examined the brain regions that were jointly active during both conditions using a univariate conjunction analysis. Subsequently, a region-of-interest (ROI) based MVPA was conducted to see if a classifier could differentiate between brain patterns corresponding to the two melodies in either condition/modality. Specifically, we focused on areas of the premotor cortex (the dorsal premotor area – PMD; the ventral premotor area – PMV), and of the auditory cortex (superior temporal gyrus – STG), since previous research shows these regions to be heavily engaged in action observation/imagery and auditory processing/imagery respectively. These regions are also engaged during the process of learning to play a melody^33^ and was in our previously study shown to display spatial patterns of activity that differ systematically between melodic pitch sequences^32^. Lastly, cross-modal classification was performed to investigate if both conditions would induce similar brain representations in the ROIs. Again, if the common coding principle were true, the spatial patterns of activity induced by a melody should be the same regardless of condition. Therefore, a classifier trained on differentiating between the melodies and their corresponding patterns of brain activity in one modality should also be able to differentiate between the corresponding brain patterns in the other modality, and vice versa.

## Materials and methods

### Participants

A total of 27 individuals participated in the study. The participants were recruited via the participant database of the Max Planck Institute for Empirical Aesthetics and via open advertisement on our homepage, using convenience sampling. The inclusion criteria required participants to be right-handed adults (> 18 years), have less than 2 years of musical training in childhood, no musical training in adulthood, no neurological or psychological disorders, and be eligible for MRI in accordance with general safety regulations. Five participants were excluded; one participant due to a technical problem with the MRI scanner, one participant did not pass the MRI safety screening, one participant chose to abort the scanning procedure, and two participants completed both sessions but were excluded due to excessive head movements (> 2 mm displacement). Thus, 22 participants were included in the final analysis (6 males, age = 30.41 ± 7.62 years). All experimental procedures followed the guidelines of The Code of the World Medical Association (Declaration of Helsinki); they were ethically approved by the Ethics Council of the Max Planck Society and the ethics committee of the Department of Medicine of the Goethe University Frankfurt (Dnr. 2017_12 and 415/17); and were undertaken with written informed consent of each participant. Participants were compensated €7 per half-hour for the behavioral study and €10 per half-hour for the MRI session, resulting in a total of €61 for each participant.

### Auditory stimuli

**Fig. 1:**
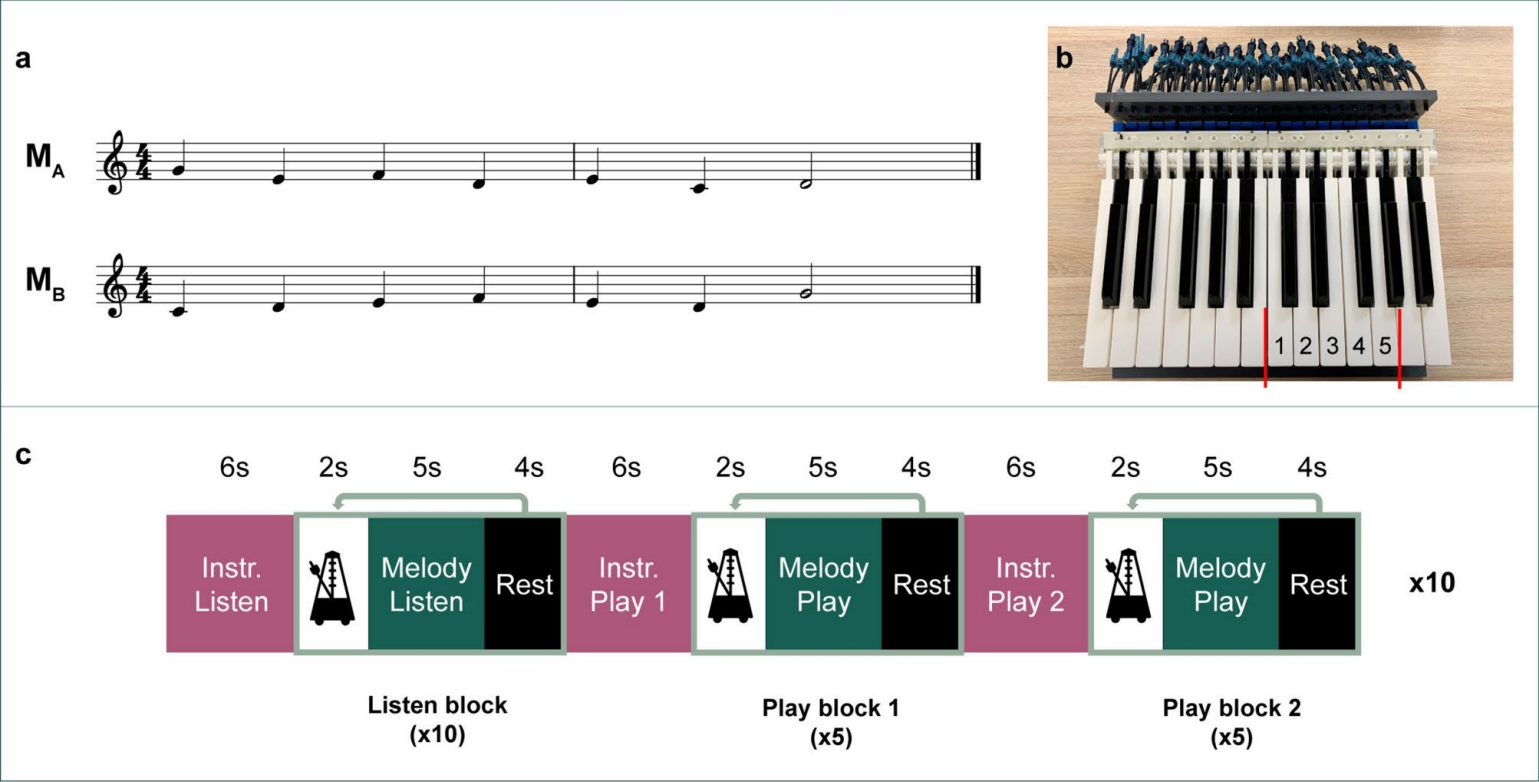
Stimuli, materials and experimental procedure. (**a**): The two melodies learned by the participants. (**b**): The MRI piano. Numbers indicate fingering starting with the thumb (1) to the little finger (5) of the right hand, matching the notes **C**, **D**, **E**, **F**, and **G**. (**c**): The experimental paradigm used in fMRI experiments. The paradigm consisted of three blocks: one listening block and two playing blocks. All blocks started with four beats of metronomes (96 bpm), followed by either melody A or B, and ended with a 4s resting period.

Two melodies were composed for this experiment. Each melody consisted of 2 bars with 7 notes from 5 pitches (Fig. 1a). Both melodies were composed to be played with the right hand only, with a pitch range from C to G so that each finger corresponded to one key. We thus avoided lateral movements of the hand in order to minimize motion artifacts. Furthermore, no pitch was repeated more than 2 times, and no more than 1 note was in the same sequential position across the melodies, in order to minimize representational overlap.

### MR-compatible keyboard

An MRI-compatible keyboard was used by the participants to play the melodies during scanning. The keyboard was based on a Kawaii digital piano that was cut down to 24 keys (2 octaves), with all metal parts replaced with plastic. The hammers were 3D-printed and weighted to match the action of the original hammers (Fig. 1b). Since this experiment did not include auditory feedback from the piano, we will not elaborate on that functionality here.

### Experimental procedure

#### Day 1 – Piano training and psychological testing

The day before the MRI experiment, participants were invited to a piano training session. Before the training session started, the participants performed the Swedish Musical Discrimination Test (SMDT) to assess their musical auditory ability^34^.

In the experimental setup, two 25-key MIDI keyboard controllers (Alesis V25) were placed next to one another to enable the participants to observe and replicate the finger movement of the instructor. The participants listened to the two melodies, which were implemented in MuseScore3™ and presented through loudspeakers. The instructor first demonstrated the finger position on one keyboard and asked the participants to place their fingers in the same way on the other. After the participants familiarized themselves with the keyboard, the instructor then played one of the melodies (one bar at a time), while the participants tried to match the finger movements. The participants were also asked to pay attention to the auditory feedback, which they heard through the speakers. When the participant had learned to play the melody independently and repeatedly (10 times in a row) without mistakes, the instructor moved on to teach the second melody. After both melodies had been memorized, the participants were asked to practice them in an alternating fashion for 10 min. The instructor then asked the participants to play without looking at their fingers, to mimic the conditions in the scanner, for another 10 min. Lastly, the instructor asked participants to imagine the melodies while playing them without auditory feedback for yet another 10 min. After this training, all participants were able to play both melodies flawlessly without looking at their fingers, without auditory feedback, and without moving anything but the right hand. In addition, the participants practiced imagining the finger movements while listening to the melodies. This familiarization process helped participants get accustomed to the tasks they would perform during the fMRI experiment. The training session lasted for 1 to 1.5 h in total. On the basis of previous studies using similar doses of training (de Manzano et al.^32^), we assumed the training duration would be sufficient to establish action-perception coupling. This assumption is also consistent with TMS studies that has shown plasticity changes in auditory-motor regions after 30–40 min of piano practice^5,7^.

### MRI scanning parameters

The MRI data were collected on a 3 T Siemens Prisma scanner with a 32-channel head coil. The functional scans were acquired using a multiband sequence with a factor of 3 with 2 mm isotropic voxels (TR = 2000 ms; TE = 30 ms; field of view (FOV) = 19.2 cm; matrix size = 96 × 96; flip angle = 90°). For each participant, we also acquired a high-resolution T1-weighted (T1w) anatomical image (1 × 1 × 1 mm^3^, TR = 2000 ms; TE = 2.12 ms; TI = 1000 ms; FOV = 25.6 cm; matrix size = 256 × 256; flip angle = 8°) and a T2-weighted (T2w) scan (1 × 1 × 1 mm^3^, TR = 1500 ms; TE = 356 ms; FOV = 25.6 cm; matrix size = 256 × 256; flip angle = 120°).

### MRI experiment

Upon arrival on the second day, the participants practiced playing the melodies for around 10 min without auditory feedback, to make sure they were able to play the melodies correctly in the scanner. The instructor then explained the tasks and demonstrated the visual instructions. During scanning, these were projected onto the screen behind the scanner and viewed through the periscope mirror system on the head coil. The visual instructions, as well as the auditory stimuli, were delivered using the Presentation® software (Version 23.0, Neurobehavioral Systems, Inc., Berkeley, CA).

In the scanner, the participants were put in supine position with the MRI-piano placed on their lap. MR-compatible noise-canceling headphones were provided (OptoAcoustics OptoActive II TM ANC) to enable presentation of the auditory stimuli. In addition, cushions were placed under the elbows of the participants to minimize fatigue and hand/arm movements.

The fMRI experiment consisted of 10 sessions (runs). Each session consisted of 2 conditions: a listening and a playing condition (Fig. 1c). During the listening condition, the participants listened to both melodies in a pseudorandomized order, with each melody presented 5 times. Each trial began with a four-beat metronome cue (96 bpm), after which the melody was played. Participants were explicitly instructed not to move their fingers during this condition, and instead imagine the finger movements as if they were playing. They were further instructed to maintain their gaze at a red fixation cross which was displayed on screen to minimize eye-movements.

The playing condition consisted of 2 blocks, each corresponding to one of the melodies. Each block began with an auditory presentation of the melody, indicating which melody the participant would be playing in that block. The order of the melodies was randomized across the two blocks, so that each melody appeared in each block an equal number of times across all participants. A four-beat metronome was then presented to indicate the tempo, upon which the red fixation cross switched to green and the participants played the melody; then the fixation cross switched back to red and there was a brief rest period. This metronome-play-rest sequence was repeated 5 times before moving on to the next block. The participants were instructed to play the piano with the right hand, without auditory feedback, while imagining the sound of the melody.

### Data analysis

#### Preprocessing of MRI data

The data were transformed from DICOM to NIfTI format using the MRIcroGL software. We then used the Statistical Parametric Mapping software package (SPM12; Wellcome Department of Imaging Neuroscience, London, UK) for MATLAB™ (The Mathworks Inc., Natick, MA, USA) to preprocess the data. The functional images were slice timing corrected and realigned to the first image of the first session. The T2w image and the fMRI images were then coregistered to the T1w image, and both T2w and T1w images were jointly segmented. All images were then normalized to the MNI standard space. For MVPA, the fMRI images were smoothed using a Gaussian kernel with a full-width-at-half-maximum (FWHM) of 4 mm, while for the univariate group-level analysis, the images were smoothed with a FWHM of 8 mm. In addition, we visually checked the functional images and used the artifact detection function in the CONN toolbox to identify potential artifacts (http://www.nitrc.org/projects/conn; McGovern Institute for Brain Research, MIT, Cambridge, MA, USA)^35^.

#### First-level univariate analysis of the fMRI data

A general linear model (GLM) combined with the standard hemodynamic response function was applied to the fMRI data to calculate beta estimates. The beta estimates were later used as input for the multivariate pattern analysis (MVPA). For the GLM, 4 regressors of interest (2 melodies × 2 conditions) were included for each session. Also included as regressors in the model were the auditory instructions before blocks (melodies), the metronome, realignment parameters, and regressors related to transient artifacts detected during preprocessing.

#### Group-level univariate analysis

The contrast images of each condition (Listen and Play) versus baseline were calculated and then entered into the second-level group analyses. To identify brain areas that were significantly activated in both conditions, we performed a conjunction analysis based on the minimum statistic test^36^. The results were corrected for multiple comparisons using a false discovery rate (FDR) threshold of *p* < 0.05^37^.

#### Multivariate pattern analysis (MVPA)

**Fig. 2:**
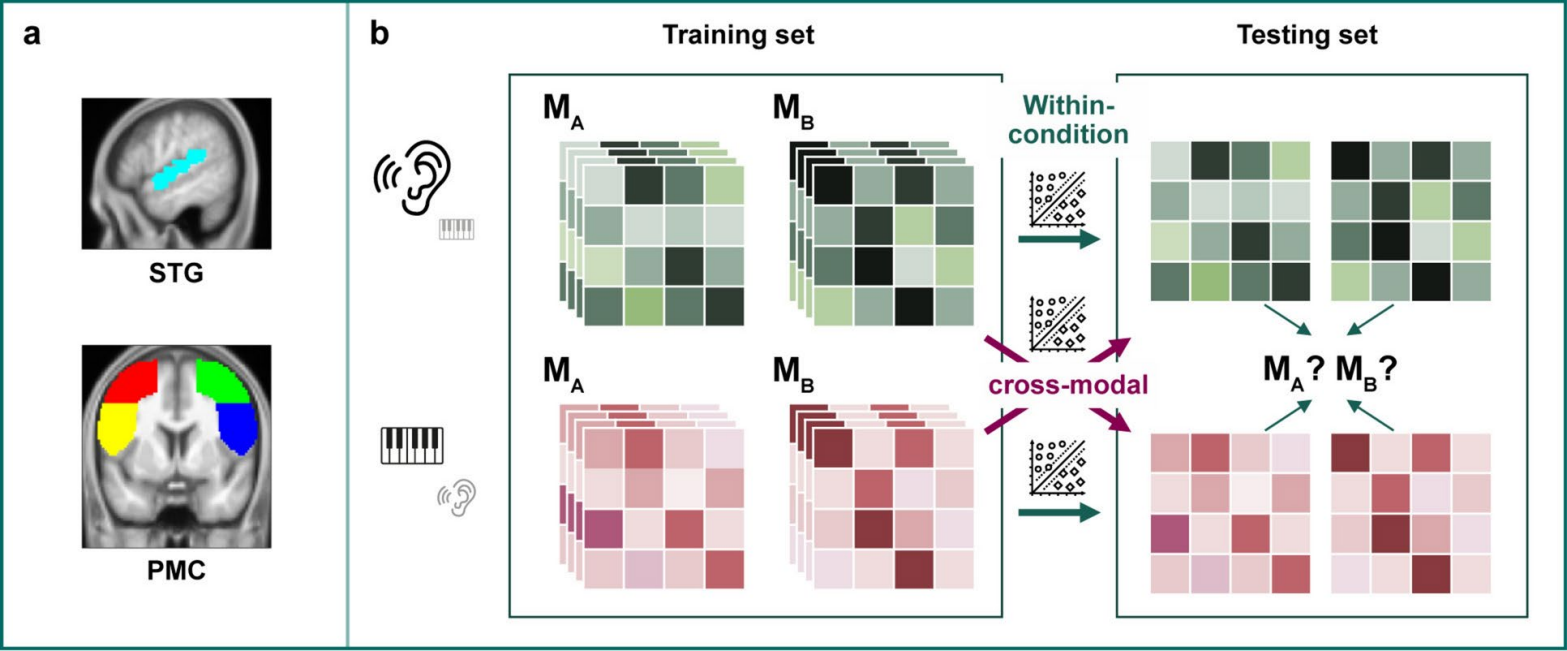
ROI-based MVPA. (**a**): Illustration of the selected ROIs: the superior temporal gyri (STG) in cyan (sagittal slice at x = − 53.0), the premotor cortex (PMC) including the PMD in red and green, and the PMV in yellow and blue (coronal slice at y = 4.6). (**b**): Illustration of the MVPA, including the within-condition MVPA and the cross-modal MVPA.

To determine whether (i) the two melodies induced sequence-specific brain representations and (ii) whether the representations would generalize across conditions, we conducted several MVPAs. These analyses were performed using The Decoding Toolbox (TDT, version 3.999E)^38^ implemented in MATLAB™. We first selected 6 a priori regions of interest (ROIs) (see Introduction), which included the left and right superior temporal gyri (left/right STG), the left and right dorsal premotor cortices (left/right PMD), and the left and right ventral premotor cortices (left/right PMV) (Fig. 2a). The STG masks were extracted from the Automated Anatomical Atlas 3 (AAL-3)^39^ while the PMD and PMV masks were obtained from the Human Motor Area Template (HMAT)^40^. The ROI masks were transformed to the native space of each participant and masked by the gray matter mask. Functional ROIs were then defined by overlaying the activation map from the conjunction analysis (uncorrected threshold of *p* < 0.001) onto these individual ROI masks.

For MVPA decoding, we used a linear support vector machine (SVM)^41^ with a leave-one-out cross-validation scheme for partitioning the data into training and test sets. Decoding was conducted in two stages. Firstly, we performed within-condition decoding, that is, the SVM was trained to distinguish between the melodies based on the voxel-wise patterns of the beta images from each condition separately. Secondly, we performed a cross-modal classification with training data from the listening condition and testing data from the playing condition, and vice versa (Fig. 2b). To determine the statistical significance of the decoding results, a permutation analysis was performed with 10,000 permutations. For the group-level results, we performed a one-tailed one-sample t-test on the individual accuracies from each ROI to test if they were significantly above chance (50%). The *p*-values were corrected for the number of ROIs using FDR. ROIs with *p*-values less than 0.05 after FDR-correction were deemed significant.

#### Auditory ability and classification accuracy

In order to evaluate if individual differences in auditory ability would relate to action-perception coupling, we performed Kendall’s Tau correlations between SMDT scores and individual classification accuracies obtained from the MVPA.

## Results

### Univariate analyses

**Fig. 3:**
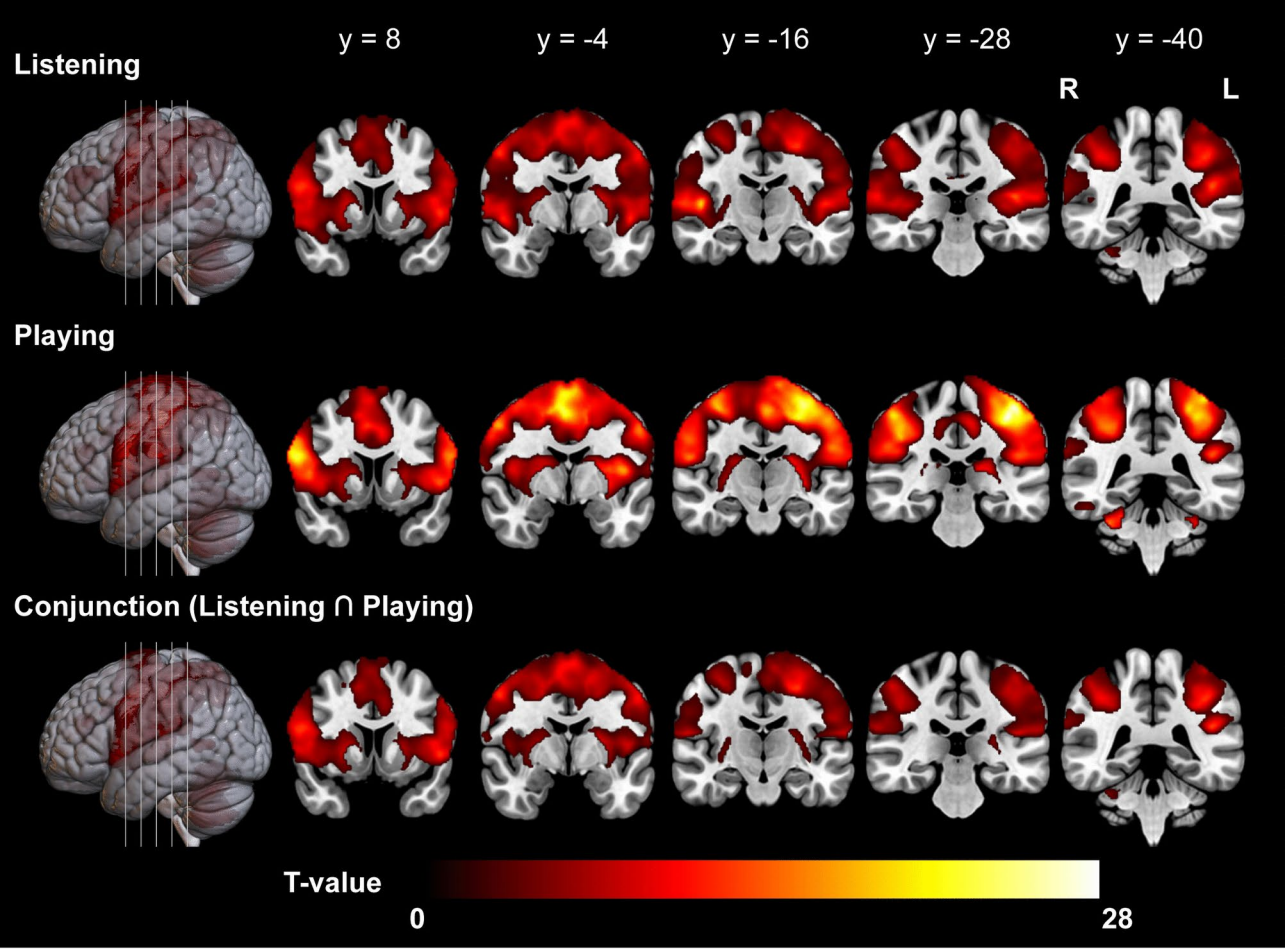
Illustration of active brain regions during the conditions. Significant activations at *p* < 0.05, FDR-corrected (y = 8, − 4, − 16, − 28, − 40).

In the listening condition, there were extensive activations across all ROIs, extending further into the parietal cortices bilaterally (Fig. 3; Supplementary Table S1). Similar regions were active in the playing condition, however with seemingly less extensive activity in the STG (Supplementary Table S2). The conjunction analysis confirmed that all of the ROIs were activated during both conditions. In addition, bilateral supplementary motor areas (SMA), primary sensory and motor cortices, and posterior parietal cortices including the superior parietal lobes (SPL) and inferior parietal lobes (IPL) were found active during both conditions (Table 1).

**Table 1:** Activation peak statistics in conjunction analysis thresholded at *p* < 0.05 (FDR-corrected). For interpretability, only clusters with a minimum of 100 contiguous voxels were reported. *k*E = the number of significant voxels in the cluster; *p*(FDR-corr) = false discovery rate corrected *p*-values.

*k*E	*p*(FDR-corr)	Peak MNI coordinate (x, y, z)	Region
31,023	0.000	[60, 4, 20]	Precentral_R
	0.000	[− 54, − 2, 42]	Precentral_L
	0.000	[− 46, − 40, 20]	Temporal_Sup_L
6475	0.000	[− 24, − 64, − 54]	Cerebellum_8_L
	0.000	[26, − 64, − 50]	Cerebellum_8_R
	0.000	[24, − 64, − 22]	Cerebellum_6_R
126	0.001	[− 46, − 58, 8]	Temporal_Mid_L
	0.008	[− 38, − 46, 10]	Temporal_Sup_L
312	0.001	[34, 40, 28]	Frontal_Mid_R
	0.002	[32, 40, 14]	Frontal_Mid_R
	0.005	[22, 36, 20]	Frontal_Mid_R

### ROI-based multivariate pattern analysis

**Fig. 4:**
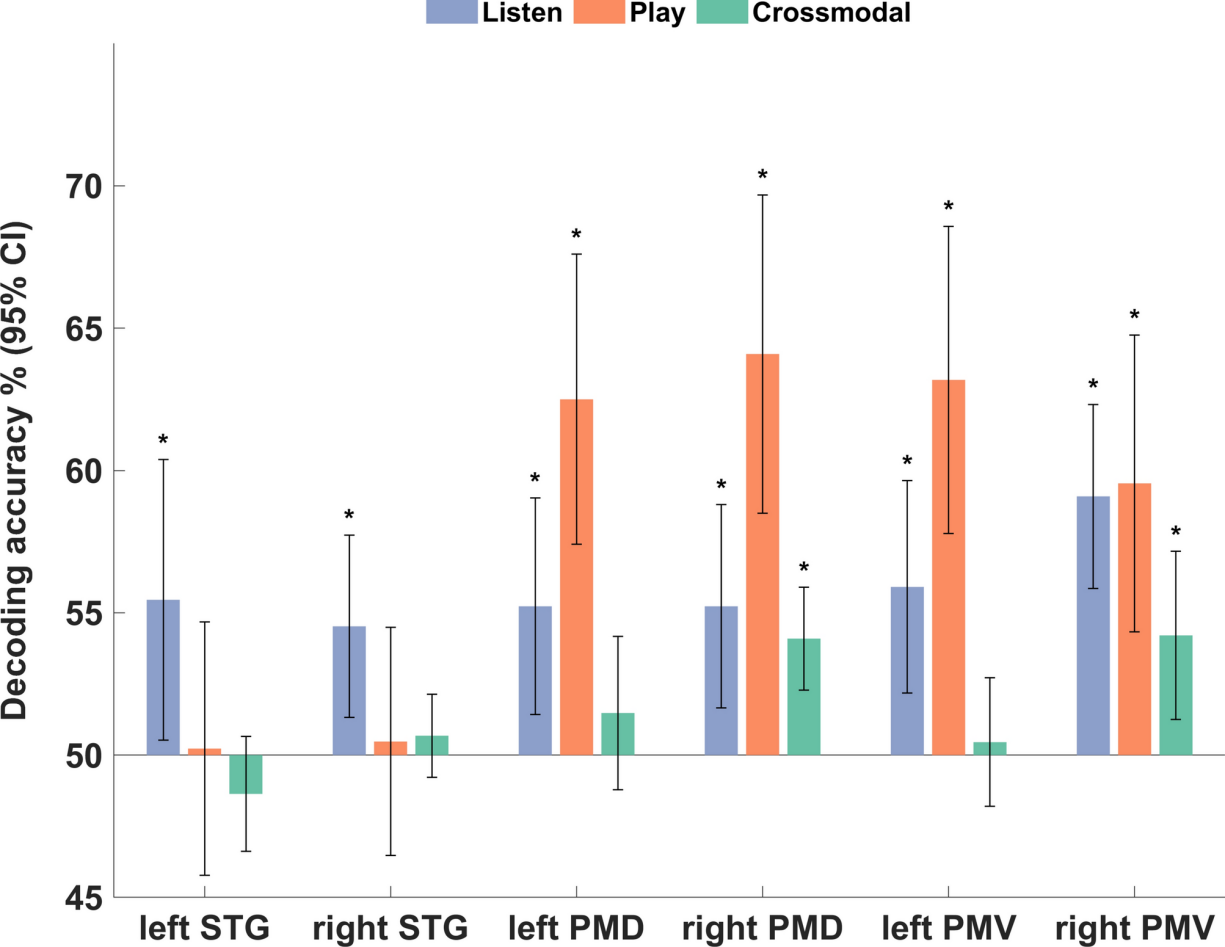
ROI-based MVPA classification accuracy results. * Significant at *p* < 0.05, FDR-corrected.

In the listening condition, all ROIs showed above-chance classification accuracies (*p* < 0.05, FDR corrected), indicating that the melodies could be successfully decoded from these regions (Fig. 4). In contrast, during the playing condition, decoding was successful in premotor regions but not in the STG. Finally, when performing the cross-modal MVPA, the right PMD and PMV showed above-chance accuracies at the group level, demonstrating its involvement in integrating the auditory and motor processing. Beyond the group level findings, permutation testing on the individual level could confirm that 10 individuals had established strong and consistent spatial patterns of activity that were similar enough to allow the machine learning classifier to train on one condition and then decode the identity of the melodies with significant accuracy in the other, from at least one of the ROIs (see Supplementary Table S3).

### Associations between auditory ability and action perception coupling

We found no evidence for an association between auditory ability (SMDT scores) and classification accuracy, neither from the within-modality MVPA of the listening condition, the playing condition nor from the cross-modal classification (Table 2).

**Table 2:** Kendall’s Tau correlation between SMDT scores and classification accuracies.

	Listening		Playing		Cross-modal	
	*τ*	*p*	*τ*	*p*	*τ*	*p*
left STG	0.00	0.818	− 0.17	0.342	− 0.08	0.625
right STG	− 0.12	0.491	− 0.08	0.643	− 0.35	0.035
left PMD	− 0.10	0.624	0.10	0.423	− 0.21	0.194
right PMD	− 0.15	0.251	0.17	0.492	− 0.09	0.565
left PMV	− 0.10	0.488	0.00	0.954	0.09	0.582
right PMV	− 0.25	0.111	− 0.16	0.228	− 0.10	0.530

## Discussion

In this study, we examined the action-perception coupling effect in non-musicians after they learned to play two melodies on the piano. According to the common coding principle, a shared neural code will represent both an action and its corresponding sensory outcome once the association between the two is established^2,4^. On this basis, we hypothesized that after learning new melodies, the perception of their sounds would evoke similar voxel-wise patterns of brain activity as the execution of the finger movements used to perform the same melody on a keyboard. In line with earlier literature we, in both conditions, found overlapping brain activity when participants listened to the trained melodies and only imagined playing, and when they were playing the piano and only imagined the sound. This overlap included motor and auditory regions, as well as extended areas in the posterior parietal lobe. MVPA further demonstrated sequence-specific representations in the bilateral PMD and PMV across both conditions, with bilateral STG reflecting distinct patterns specifically during the listening condition. Most importantly, we found support for our main hypothesis, showing that brain activity patterns in the right PMD and right PMV could be generalized across modalities in many specific individuals and at the group level, as indicated by the cross-modal classification results. There were individual differences in the classification accuracy, indicating variability in auditory-motor integration between participants; however, this variability could not be attributed to individual differences in auditory ability. Instead, it appears that other factors were at play, and we suggest that a longer training period may have facilitated the establishment of more robust sensorimotor associations between pitch sequences and finger movements across the sample. We elaborate on these findings below.

### Univariate analysis

Action observation, imagery, and execution recruit largely overlapping sets of brain regions, as revealed by previous research^21,42,43^. These areas include the PMD, PMV, SMA, IPL, and SPL. Furthermore, in the context of perceiving an action associated with sound or auditory imagery, the STG is also involved^11,44–46^. Our results from the conjunction analysis support these findings, showing joint activation in the aforementioned regions during both conditions. However, it is important to highlight that although the regions were jointly activated, univariate analysis does not provide evidence that the regions represented the melodies equally during both conditions. On the contrary, the multivariate analyses of patterns of activity in auditory-motor areas point to interesting differences between listening with motor imagery and playing with auditory imagery.

### Within-condition MVPA

A primary purpose of these multivariate analyses was to investigate whether there were sequence-specific representations of pitches/finger movements in the ROIs. Classification within the listening condition essentially replicated our previously reported findings^32^, showing above-chance classification accuracies in all ROIs. For the playing condition, the classification accuracy was significant only for the premotor regions. One explanation for the non-significant classification in auditory regions could be interference between auditory imagery during playing and scanner noise, which is naturally a limiting factor and perhaps even more so in non-musicians.

### Cross-modal MVPA

Cross-modal classification allowed us to examine the formation of a common representational code between listening (while imagining playing) and playing (while imagining listening) to the melodies. Out of the 6 ROIs selected, we found that decoding across conditions and across the sample was successful in the right PMD and PMV, suggesting that common sequence-specific patterns were formed in these regions. These results support the common coding principle; that the association between an action sequence and its corresponding sensory outcome is established in the form of a shared brain representation, or more specifically, a shared spatial pattern of brain activity. Notably, previous studies have primarily applied cross-modal classification to single actions. Our study extends this approach to motor sequences, which demonstrates that common codes can also be formed for more complex action patterns.

The presence of shared representations between action and perception in the premotor cortex aligns with previous studies, which highlighted the role of this area in auditory-motor coupling^26,32,47–49^. Located at the interface between the primary motor cortex and STG, the premotor cortex integrates inputs from motor and sensory aspects^20^. The right PMD and PMV, in particular, appear to be crucial for this integration, as they exhibit consistent activation patterns when the participants perform and perceive the same melody. Although it may seem surprising that the right hemisphere showed more prominent results than the left, given that only the right hand was used by the participants throughout the task, previous studies have illustrated the recruitment of ipsilateral motor cortex during movements that require higher task demands^50,51^. Wiestler and colleagues also reported effector independent representations in these regions^52^. Additionally, the right hemisphere has been shown to be more involved in musical processing, which may further contribute to its role in auditory-motor integration^53–55^. While it remains unclear whether these findings are due to lateralization, our findings suggest that the right hemisphere plays a more significant role in this process.

In contrast, we did not find cross-modal representations in the left PMD and PMV across the sample, despite observing within-condition sequence-specific patterns in these areas during both the listening and playing conditions. In other words, while the left premotor cortex was engaged in processing the auditory and motor sequences, the sequence representations were condition-specific rather than shared. Notably, even though action and perception can be coupled, they are not usually confused, and our findings indicate that distinct and shared codes are maintained in parallel, by lateralizing the different forms of representation. This might be an interesting avenue for further research on the integration and segregation of action and perception.

Although the STG was jointly activated during both conditions, as shown by the conjunction analysis, no cross-modal representations were present. This might be related to the results from the within-condition MVPA, where the classifier did not perform above-chance during the playing/imagine-sound condition. As suggested previously, this could be related to auditory imagery being more inconsistent, perhaps in part due to the scanner noise, resulting in lower classification performance. Consequently, this variability would also tend to reduce cross-modal classification accuracy.

Individual differences across regions were observed, as shown in Supplementary Table S3. While we found strong and significant evidence for cross-modal representations at the individual level in 10 participants, there were some differences with regard to the specific ROIs. This suggests that many participants indeed formed integrated action-sound representations during training, which were used during the performance of both conditions, but also that there were individual differences in the spatial location of these representations. One potential source of variability could be that the more fine-grained functional organization of brain regions involved in the processing of music depends on learning and plasticity related to previous musical experiences and music listening habits.

Given this variability, an interesting question for future studies is to see if common auditory-motor codes can also form in other regions. Although we found extensive overlaps in brain activity between listening and playing conditions, we found the “common code” mostly in the right PMD and PMV. Previous studies have demonstrated that 20–30 min of piano training in naïve participants could lead to plasticity changes in the brain or intracortical facilitation^7,56^. At the same time, during the learning process, the brain is still reorganizing, and therefore the sequence representations may vary across time during the experiment^57,58^. Indeed, previous studies indicate that sequence-specific representations may be more robust after longer periods (days or weeks) of training^30,31,59^. Thus, this study, with its limited training dose and musical material, could be viewed as a proof of concept that future work could extend on.

To this date, not many studies have applied cross-modal classification within auditory and motor domains^26^. The majority of studies that have reported successful cross-modal decoding focused on the relationship between visual and motor representations^28,60^. In addition, only a few investigations have specifically addressed sequential learning. Consequently, numerous factors might conceivably influence cross-modal classification that require further investigation. As mentioned previously, the individual differences in classification accuracy observed in this study suggest that the presence of such influencing factors. Future studies could investigate the relationship between factors such as training dose, personal characteristics, and previous personal experience on the formation of action-perception coupling. This study was limited with regard to such information. Exploring similar factors could provide valuable insights into the neural mechanisms underlying musical learning and skill learning in general.

Despite the complexities outlined above, a fundamental question arises as to whether the common coding principle fully explains the action-perception coupling effect. While several studies have supported this notion, it is still possible that during the initial stage of the learning process, there are separate codes for action and perception. As famously proposed by Donald Hebb, synaptic connections between neurons may increase in strength when they are repeatedly activated simultaneously, so that initially independent neural circuits eventually form larger integrated assemblies that control behavior^61^. Along these lines, it could be speculated that although different coding principles are used for sensory and motor information at the initial learning stage, such modality-specific representations can through repeated coactivation and plastic adaptations in sensorimotor brain networks eventually become unified into a more abstract code that facilitates or even automates bidirectional associations.

## Conclusions

In summary, our study demonstrates, firstly, that sequence-specific representations of complex acquired skills and their sensory consequences are possible to establish via short-term training. Specifically, melody-specific patterns of neural activity were found in bilateral PMD and PMV in both listening and playing conditions, while STG only showed distinct patterns in the listening condition. Secondly, we found evidence for cross-modal representations in the right PMD and right PMV, suggesting that these regions play an important role for integrating action and sound, including tasks involving mental imagery. Finally, analyses of single participants indicate individual variation in cognitive strategies and the spatial localization of relevant neural processes. Important questions for future studies will be to address the implications of individual variation in neural coding, as well as the question whether common coding can be established in different brain regions as action-perception coupling is strengthened during prolonged training.

## Supplementary Information

Supplementary Information.

## Data Availability

The datasets generated during and/or analyzed during the current study are available from the corresponding author on reasonable request.
