# Supplementary material for: Common brain representations of action and perception investigated with cross-modal classification of newly learned melodies
Source: Sci Rep. 2025 May 12;15:16492. doi: 10.1038/s41598-025-00208-x (PMC12069664; doi:10.1038/s41598-025-00208-x)
Supplement: Supplementary file 1 — Supplementary Information. [file 41598_2025_208_MOESM1_ESM.pdf]

Supplementary Materials for

Common brain representations of action and perception investigated with cross-modal classification of newly learned melodies

Yu-Hsin (Fiona) Chang[1, 2, *], Fredrik Ullén[1, 2, †], and Örjan de Manzano[1, 2, †]

[*]Corresponding author. E-mail: yu-hsin.chang@ae.mpg.de

[†]shared senior authorship

Table S1. Listen condition (thresholded at peak-level *p* < 0.05, FDR-corrected). For interpretability, only clusters with a minimum of 100 contiguous voxels are reported. *k*E = the number of significant voxels in the cluster; FDR-corr = false discovery rate corrected *p*-values.

k_E	*p*(FDR-corr)	peak MNI coordinate (x, y, z)	Region
42745	0.000	[48, -16, 6]	Heschl_R
	0.000	[-48, -24, 8]	Temporal_Sup_L
	0.000	[58, -20, 4]	Temporal_Sup_R
7085	0.000	[-24, -64, -54]	Cerebellum_8_L
	0.000	[26, -64, -50]	Cerebellum_8_R
	0.000	[24, -64, -22]	Cerebellum 6_R
785	0.000	[-36, 32, 26]	Frontal_Inf_Tri_L
	0.002	[-34, 50, 28]	Frontal_Mid_L
	0.017	[-34, 44, 12]	Frontal_Mid_L
915	0.000	[36, 38, 28]	Frontal_Mid_R
	0.001	[36, 40, 16]	Frontal_Mid_R
	0.002	[36, 44, 2]	Frontal_Mid_R

Table S2. Playing condition (thresholded at peak-level $p < 0.05$, FDR-corrected). For interpretability, only clusters with a minimum of 100 contiguous voxels were reported. kE = the number of significant voxels in the cluster; FDR-corr = false discovery rate corrected p-values.

k_E	p(FDR-corr)	peak MNI coordinate (x, y, z)	Region
40574	0.000	[-38, -28, 50]	Postcentral_L
	0.000	[-44, -28, 58]	Postcentral_L
	0.000	[60, 6, 20]	Precentral_R
11129	0.000	[-22, -54, -22]	Cerebellum_6_L
	0.000	[18, -60, -46]	Cerebellum_8_R
	0.000	[2, -64, -12]	Vermis_6
474	0.000	[24, 34, 18]	Frontal_Mid_R
	0.000	[30, 40, 16]	Frontal_Mid_R
	0.000	[34, 40, 24]	Frontal_Mid_R

Table S3. Individual cross-modal classification accuracies. *Significant at $p < 0.05$ after permutation analysis (n = 10000).

Participant	left STG	right STG	left PMD	right PMD	left PMV	right PMV
1	60	52.5	55	52.5	45	50
2	50	52.5	60	62.5*	45	52.5
3	57.5	52.5	50	47.5	50	52.5
4	50	47.5	47.5	52.5	45	45
5	40	50	50	50	50	37.5
6	47.5	50	50	60	47.5	67.5*
7	60*	52.5	47.5	55	50	62.5*
8	45	47.5	72.5*	57.5	62.5*	57.5
9	42.5	60	45	50	50	47.5
10	52.5	50	50	50	65*	57.5
11	52.5	52.5	52.5	57.5	57.5	52.5
12	47.5	55	62.5*	55	47.5	47.5
13	52.5	55	47.5	55	55	67.5*
14	50	50	35	47.5	42.5	50
15	40	50	45	42.5	52.5	55
16	45	50	50	50	50	60
17	50	45	57.5	60	60	62.5*
18	47.5	40	50	55	40	55
19	45	55	60	57.5	42.5	65*
20	42.5	52.5	45	52.5	52.5	37.5
21	50	45	47.5	62.5*	50	60
22	42.5	50	52.5	57.5	50	50
